# The EBRAINS NeuroFeatureExtract: An Online Resource for the Extraction of Neural Activity Features From Electrophysiological Data

**DOI:** 10.3389/fninf.2021.713899

**Published:** 2021-08-26

**Authors:** Luca L. Bologna, Roberto Smiriglia, Dario Curreri, Michele Migliore

**Affiliations:** Institute of Biophysics, National Research Council, Palermo, Italy

**Keywords:** electrophysiology, data analysis, online resources, neural models, EBRAINS

## Abstract

The description of neural dynamics, in terms of precise characterizations of action potential timings and shape and voltage related measures, is fundamental for a deeper understanding of the neural code and its information content. Not only such measures serve the scientific questions posed by experimentalists but are increasingly being used by computational neuroscientists for the construction of biophysically detailed data-driven models. Nonetheless, online resources enabling users to perform such feature extraction operation are lacking. To address this problem, in the framework of the Human Brain Project and the EBRAINS research infrastructure, we have developed and made available to the scientific community the NeuroFeatureExtract, an open-access online resource for the extraction of electrophysiological features from neural activity data. This tool allows to select electrophysiological traces of interest, fetched from public repositories or from users’ own data, and provides *ad hoc* functionalities to extract relevant features. The output files are properly formatted for further analysis, including data-driven neural model optimization.

## Introduction

Data analysis of electrophysiological traces is at the core of a wide range of studies in the neuroscientific field. On the one hand, the characterization of neural activity is a necessary step for understanding the behavior of individual cells (Petersen, 2017) and ensembles of neurons (Shahaf and Marom, 2001) and its correlates: the sensory, cognitive and behavioral counterpart of an electrophysiological measure (Rizzolatti and Craighero, 2004; Johansson and Flanagan, 2009). On the other hand, experimental findings are instrumental for the construction of detailed neural models, able to robustly and accurately reproduce the observed activity (Migliore et al., 2018). More specifically, the broader is the range of electrophysiological features extracted from the experimental observations (e.g., action potential amplitude and width, Inter Spike Intervals, resting potential, etc.), the more precise and biophysically detailed is the computational model based on those features.

Nowadays, the computational neuroscience community can rely on an increasing number of online, freely accessible tools and platforms for neural model building and collaborative sharing. Well-established, open and free simulation environments such as NEURON (Hines and Carnevale, 1997), Nest (Gewaltig and Diesmann, 2007; Eppler et al., 2009), and Brian (Goodman and Brette, 2008; Stimberg et al., 2019) are being constantly maintained and upgraded with new functionalities and fervent user communities contribute to their improvement with useful feedbacks. In addition, several platforms are available to the community, for model sharing, testing, running and publication, that are increasingly embodying an open access approach. For example, ModelDB^1^ (Branco et al., 2010; McDougal et al., 2015, 2017) has become a reference portal for computational neuroscientists aiming at archiving and sharing their work. The platform allows to: (1) upload any kind of neural models -which must be provided with *ad hoc* scripts to test their correct functioning; (2) label them; (3) run them on a dedicated simulation platform; (4) annotate, for each model, the relevant and/or relative scientific publications. Another collaborative public online resource is OpenSourceBrain (OSB) (Gleeson et al., 2019). OSB provides visualization, simulation, analysis, and sharing tools and services for standardized neural models. Thanks to the model description languages NeuroML (Gleeson et al., 2010; Cannon et al., 2014) and PyNN (Davison et al., 2009), models are defined in standardized formats that are automatically read by OSB for simulation and model details visualization. With respect to scientific data sharing, a number of platforms have been recently created (e.g., DANDI,^2^ Zenodo,^3^ Dryad,^4^ FigShare^5^) and the Neurodata Without Borders^6^ (Teeters et al., 2015) ecosystem is fostering the development of tools and services for data analysis and visualization^7^ ^,8^. Additionally, data analysis toolkits for electrophysiological measures are available -such as the Elephant software (Denker et al., 2018)- and scientific languages and environments (e.g., MATLAB, R, Python) already provide the necessary libraries for data processing (including analysis and visualization). Furthermore, the Allen Institute^9^ provides comprehensive datasets of neuroscientific images and electrophysiological data that users can explore, download and analyze thanks to a dedicated Software Development Kit (SDK). Also, the Knowledge Graph (KG) of the EBRAINS research infrastructure^10^ is promisingly building a reference portal for data -as well as models and software- produced in the framework of the Human Brain Project (HBP) (Amunts et al., 2016) and converging into the EBRAINS European research infrastructure.^11^ Unfortunately, to the best of our knowledge, none of the existing platforms allows automated online, point-and-click services for electrophysiological feature extraction. Indeed, the Allen Institute portal provides feature values on sets of neural activity data, but the number of features is limited and the extraction process cannot be performed online but only after downloading the data and launching the appropriate routines locally (via the above-mentioned Allen SDK).

In order to address these challenges, we built a web-based resource, the EBRAINS NeuroFeatureExtract (NFE),^12^ that not only allows to extract electrophysiological features from neural activity data, but also provides users with a user-friendly point-and-click interface for uploading their own data and feed them to the extraction workflow. The web application leverages the Electrophys Feature Extract Library (eFEL) and the BluePyEfe library (see section *Methods/eFEL* and *Methods/BluePyEfe*) and provides result files properly formatted for further analysis (e.g., neural model optimization via the BluePyOpt optimization library, see section “Methods”).

## Methods

### Overview

The EBRAINS NeuroFeatureExtract consists of a full stack web-based application implemented via the Python-based Django web framework^13^ and deployed on a dedicated Virtual Machine (VM) hosted on the CINECA supercomputing center^14^ and accessible/configurable through the OpenStack interface.^15^ The VM configuration presents 24GB RAM and 8 VirtualCPU (VCPU).

The web application consists of a frontend (client-side) and a backend (server-side) components that, despite residing on the same VM, are logically separated and communicate through dedicated Representational State Transfer (REST) Application Programmer Interface (API) calls. The frontend provides a user-friendly GUI that allows an easy point-and-click interaction with the tool functionalities and is implemented via HTML, Javascript, and CSS code. The backend serves the frontend requests by running the data fetching and management, the feature extraction operations and the result files creation; it is entirely developed in Python.^16^ The server manages the requests by creating dedicated folder trees, based on the file system organization (i.e., no sandbox process is spun off; the system resources are used instead), any time the NFE is accessed and initialized. All data management operations are performed by a system user with limited access and privileges to the VM resources, in order to limit security vulnerabilities. Finally, data and results are periodically removed (see section *Methods/File formats*).

The web server fulfilling the client requests is NGINX^17^ used in conjunction with the uWSGI web interface^18^ and the communication protocol used for accessing the web application is the Hypertext Transfer Protocol Secure (HTTPS). The web application is part of a larger Django project that includes a web resource called Hodgkin-Huxley Neuron Builder (see section *Usage/Extracted features*) in which the NFE is integrated. The code is available to the scientific community (Gleeson et al., 2017) on GitHub, under the LGPLv3 license.^19^

### eFEL

The NFE core operations are based on the eFEL library, which provides the computational engine upon which the analysis of the electrophysiological traces relies. The software package is available, for command line installation, on PyPi,^20^ and its code and documentation are freely accessible on GitHub^21^ and ReadTheDocs,^22^ respectively. The core of the eFEL consists of a set of C++ libraries, which perform the low-level computations. This layer is exposed to the user via a wrapper built through a set Python API functions. No GUI is provided to the user.

The eFEL library provides the scientific community with a tool allowing the automatic extraction of electrophysiological features from *in vivo*, *in vitro*, and *in silico* neural activity recordings. The library allows to select among more than one hundred different features which are grouped by the following three categories: (1) Spike event features; (2) Spike shape features; (3) Voltage features.

Overall, spike event features provide information on the timing of the detected action potentials (AP, or spikes), such as the inverse of the timestamp of the first and last spikes, the inverse of the first to fifth Inter Spike Intervals (ISI, namely the time between two consecutive spikes) and the spike half width. Spike shape features provide information on the AP shape, such as the AP widths and heights and the AP mean amplitude. Finally, the voltage features report voltage related indices, such as the voltage base value (i.e., the membrane resting potential) and the maximum voltage during a stimulus.

In order to familiarize with the tool and to verify the correctness of the computed results, users can run, on a local machine, the unit tests included in the eFEL Github repository.

### BluePyEfe

The output of the NFE consists of a set of .*pdf* and text (i.e., .*json* and .*txt*) files that display the neural activity and stimulus traces, report statistics on the extracted features and contain recording protocol information properly formatted for further processing (see section *Usage/Results*). Since the eFEL library APIs return the computed features in the form of Python value arrays and delegate to the user further *post hoc* analysis and data formatting and printing, we used an eFEL-wrapper Python package named BluePyEfe, in order to generate a consistent set of output files. This library is entirely built in Python and is available for installation on PyPi.^23^ The source code of the library is also publicly available on GitHub.^24^

The BluePyEfe workflow consists of three main steps: (1) the electrophysiological data are read and appropriately grouped and organized in Python dictionaries; (2) the features selected by the user are extracted and their mean values computed; the computation of the average values is first performed on data referring to the same cell; then, the individual cell mean values are averaged, in order to infer the global behavior of the provided data; (3) several output files are generated that report: the above-mentioned mean values (per cell and overall), the plot of the raw stimulus and membrane voltage traces, the values of individual feature extracted (see section *Usage/Results*).

As mentioned for the eFEL library, also the BluePyEfe includes a series of unit tests that the users can start from for familiarizing with the tool and verifying the generated output.

### File Formats

The NFE allows the users to select data from a public dataset (currently provided by HBP members and collaborators but not limited to these contributors) and/or to upload their own data (see section *Usage/Upload*).

The platform is as much as possible agnostic with respect to the data format of the files the user can upload and we are aiming to extend the data upload functionality to a set as large as possible (e.g., all electrophysiological data formats accepted by the BluePyEfe and the Neo package; Garcia et al., 2014). Currently, the file formats accepted for upload are .*abf* (Axon Binary File), which must be uploaded together with a metadata file in .*json* format) and .*json* (i.e., text files with special formatting). The latter file type is structured in such a way to guarantee a fast access to both recorded data and related metadata. In the .*json* data file, the information is structured into dictionaries where the membrane voltage values and the timing information on the stimulus (i.e., start and end time of the delivered current) are grouped by stimulus amplitudes. A detailed description of the accepted file formats is available in the NFE’s Guidebook.^25^

The public data available in the NFE are currently stored in a dedicated Object Storage container at CSCS (Swiss National Supercomputing Centre) in .*json* format and are accessible from the NFE in read-only mode. A subset of the data collection contributed by HBP members and collaborators are also available (in their original format), stored, categorized and indexed in the KG, which allows public contribution from the broader neuroscientific community and is expected to become a standard reference for data publication and sharing. A tight integration between the NFE and the KG is currently under development and will disclose to the NFE’s users a continuous and seamless access to a plethora of electrophysiological data (see section “Discussion”).

## Usage

### Overview

**FIGURE 1:**
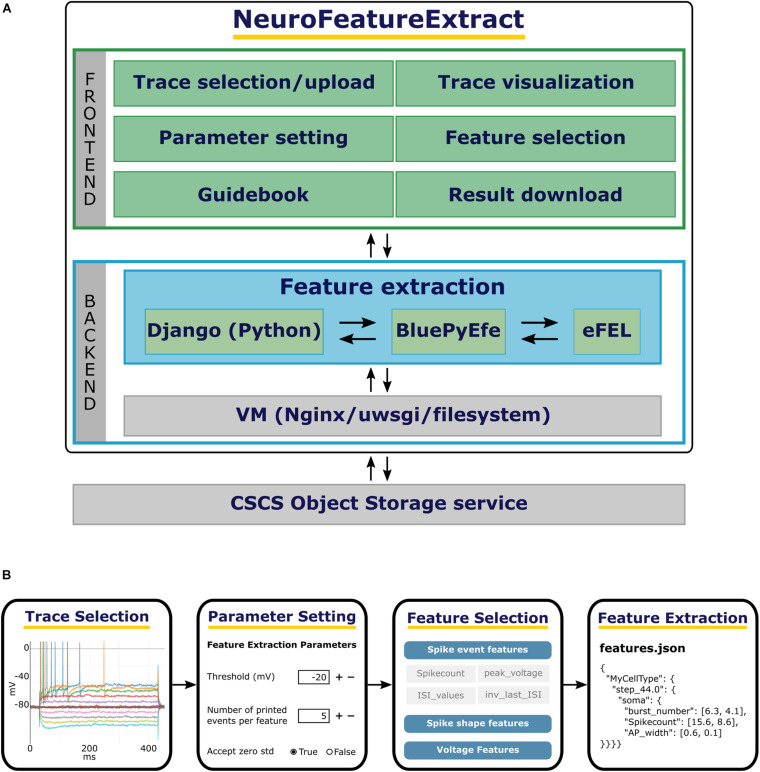
The NFE architecture and workflow. **(A)** From top to bottom: the NFE’s frontend provides a point-and-click interface to execute the trace selection, upload, visualization as well as the parameter setting and feature selection operations. The backend performs the feature extraction on the selected traces via the BluePyEfe library which in turn leverages the eFEL software. The traces made available to the users are stored in a CSCS container. **(B)** From left to right: individual traces are selected from the public dataset or from the files uploaded by the user; if needed, the extraction parameters are appropriately set; the features of interest are selected and, finally, the result files are downloaded and used for further analysis or model optimization.

The NFE GUI consists of a number of point-and-click user friendly HTML pages that guide the user through the complete feature extraction workflow (see Figure 1). On the Overview page, a short introduction to the tool is given and a quick-tour section can be unfolded with basic guidelines to the use of the web application. On the right side of the header panel, three icons are provided, which redirect to the NFE’s homepage, the NFE’s Guidebook and a video tutorial, respectively.

### Data Selection

The “Trace selection” page is divided into three sections. The top panel allows to configure a number of extraction parameters, which will be fed to the BluePyEfe instance on the backend server side. The central section allows to select the electrophysiological traces to analyze. The current list contains public data and is constantly updated with the neuroscientific community contributions. The bottom section allows the users to upload and use their own electrophysiological data in the feature extraction process.

#### Feature Extraction Parameters

Three feature extraction parameters can be currently set by the user.

**FIGURE 2:**
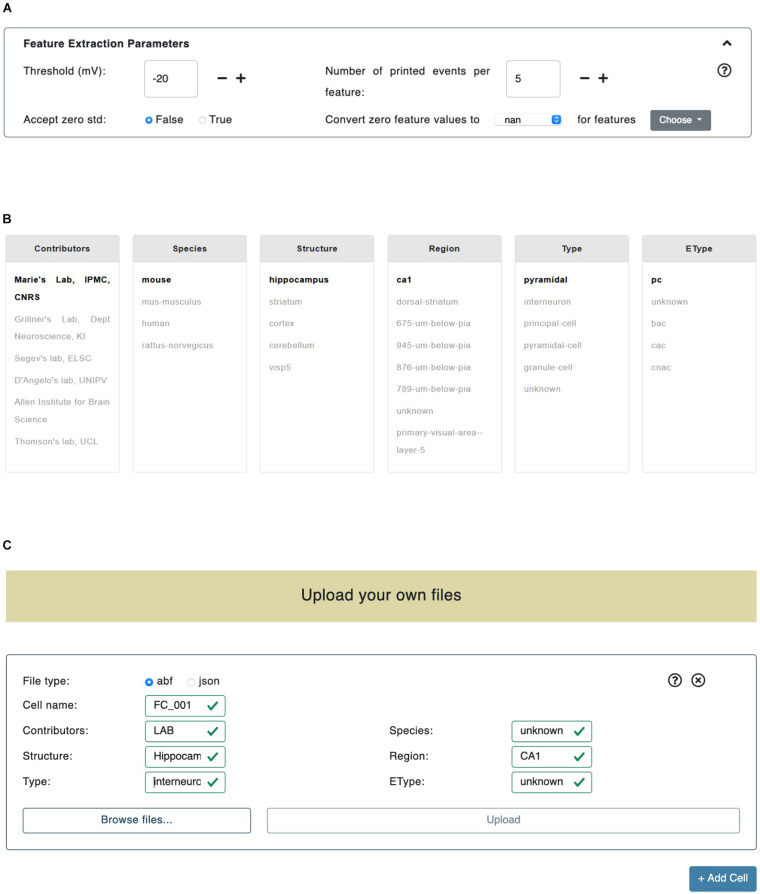
Trace selection page. **(A)** In the “Feature Extraction Parameters” panel the users can set the parameters adopted in the feature extraction process. **(B)** The data files provided by HBP members and collaborators are categorized by metadata (e.g., Contributors, Species, Structure, etc.). By clicking on a specific key, all the remaining fields are automatically updated (i.e., their color is changed) in order to reflect the available choices left to the users. **(C)** The upload panel allows to upload either .*abf* or .*json* files. Any number of panels can be added (through the bottom-right “+Add Cell” button): all the files uploaded in a single panel are considered as recorded from the same cell.

The *Threshold (mV)* parameter is the membrane potential threshold adopted for action potential detection and is particularly useful when a hard threshold (usually set to -20 or -30 mV) is not able to detect all the recorded action potentials (see section “Results”). The *Accept zero std* parameter is a flag (i.e., can be either True or False); if set to False it will collect (for final averaging and printing, see section *Methods/BluePyEfe*) only mean feature values that present either a mean value of zero or a standard deviation value greater than zero; otherwise, if set to True, all mean values (different than *not a number* -or “*nan*”) will be taken into account. We introduced this functionality in the BluePyEfe (and exposed it through the NFE GUI) because, while in general mean and standard deviation values make sense (and are strictly defined) when computed from a pool of samples, in specific cases, the users might want to account for features (e.g., the Spikecount) extracted from individual traces, for analysis purposes. Finally, the *Convert zero feature value* applies a post extraction data correction on the computed feature values. The user is provided with two dropdown menus on the GUI. The leftmost menu presents two options: *nan* and *stim_end* while the rightmost one displays a list of selectable features (see Figure 2A). When either *nan* or *stim_end* is selected, the values of the checked features are converted to *nan* or to the stimulus end time, respectively, if their value is zero after the feature extraction has been performed. This functionality is only available for a subset of features and is instrumental in the fine tuning of the model optimization process that the user might want to undergo by adopting the NFE result files (see section *Usage/Extracted features*).

#### Datasets

The central section of the *Trace selection* page allows the user to select the electrophysiological data to be used for the feature extraction from a pool of public data files. The recordings are grouped by six criteria: (1) Contributors (e.g., contributor names); (2) Species (e.g., human, rats, mice); (3) Structure (e.g., hippocampus, cerebellum, striatum); (4) Region (e.g., CA1, dorsal-striatum, primary visual area); (5) Type (e.g., interneurons, granule-cells, principal-cells), and (6) EType (e.g., continuous adapting cells or *cAC*, continuous non-adapting cells or *cNAC*) (see Figure 2B). When a value, belonging to any of the above-mentioned categories, is selected, the metadata of the data files fulfilling the selection are parsed in order to find any match with the remaining categories. The entries, in any category other than the most recently selected, that are not found in the metadata, are grayed out in the panel. Also, if after a first selection in a given category, only one match is found in another selection box, the latter is set to bold. This allows to guide the users in their further selections. An example of this logic is given in Figure 2B, where the *Contributors/Marie’s Lab, IPMC, CNRS* entry has been selected. The datasets are continuously being updated and tightly linked to the KG (see section *Methods/File formats*). We encourage the NFE users to register their data in the KG and, successively, make them publicly available in the dedicated NFE data section. The KG data registration is implemented through an agile curation process and is supported upon request at support@ebrains.eu.

#### Upload

In addition to the selection of contributed electrophysiological traces, users can also upload their own data through the NFE GUI. Uploaded files are inserted through dedicated panels which are dynamically created/deleted via *ad hoc* buttons (see Figure 2C). Once a new panel is displayed, the type of data that will be uploaded through that panel has to be specified (at the moment two formats are available: .*abf* and .*json*, see section *Methods/File formats*). All the files uploaded via a specific panel will be considered as referring to (namely, being recorded from) the same cell: this will allow to group the statistics of cell-specific features into cell-specific files (see section “Results”). In order to specify the type of cell the uploaded files refer to, a number of fields must be compulsorily filled out (e.g., Cell id, Species, Structure, Region, etc.). The neural traces uploaded by the users and the public ones can be inclusively selected for feature extraction. The files uploaded by the users are only temporarily stored on the server hosting the NFE, as this is a technical requirement for the execution of the workflow. At execution time, they are not accessible by any user other than the one who uploaded them in that every feature extraction process has its own unique session variables that allow to create a dedicated folder structure in which data, results and temporary files are stored (see section “Overview”). All uploaded files, as well as results and temporary data, are deleted from the server 2 h after they have been created (this time window is suitable to give the users enough time to complete the workflow) and are not anymore retrievable by either the NFE or data owners (a new upload is required if the users want to perform further analysis—or repeat previous ones—on the same data).

#### Trace Visualization and Selection

**FIGURE 3:**
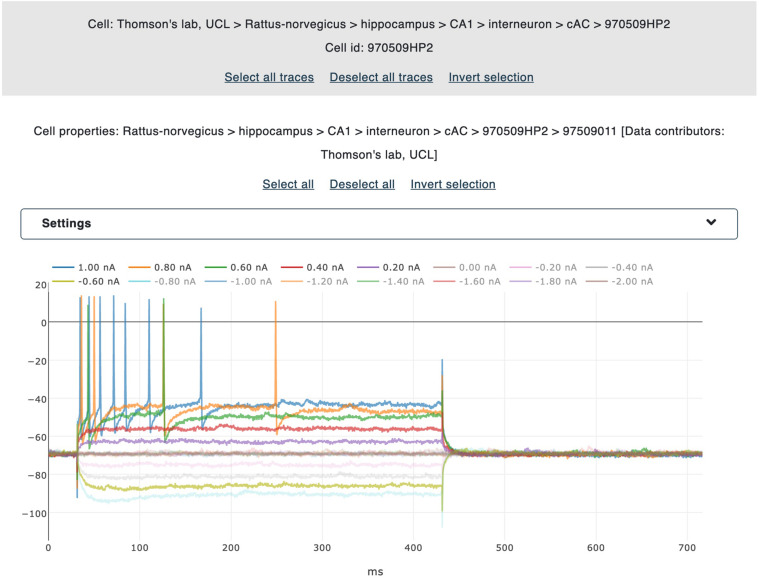
Trace selection panel. The traces belonging to individual files are displayed (together with the corresponding metadata) in interactive panels. By clicking on the legend labels, the user selects the traces to be gathered for the feature extraction. All selected traces are highlighted for ease of visualization. Data from Migliore et al. (2018).

A user-friendly interface is available for the selection of the electrophysiological traces to be processed. All the traces belonging to the same recording are collectively visualized in the same plot, which is in turn embedded into a dedicated panel showing the name of the relative file and the property of the cell from which the neural activity has been recorded. Different colors are adopted for different traces and individual trace labels are coupled with the stimulus amplitude (and unit) delivered during the signal acquisition. By clicking on a label, the corresponding trace is selected and collected for further processing (i.e., the feature extraction procedure, see Figure 3). For every recording, a voltage correction value can be set in case a holding voltage has been applied during the experiment. When applied, all the traces belonging to the same file are shifted by the inserted value. This functionality is part of the BluePyEfe software library and has been implemented (and exposed in the NFE) because, in many cases, recordings are carried out and stored with a membrane voltage shifted to zero (for experimental reasons) and this implies that a standard action potential detection threshold (of about -20 or -30 mV) might fail in detecting the recorded spikes.

### Feature Selection

**FIGURE 4:**
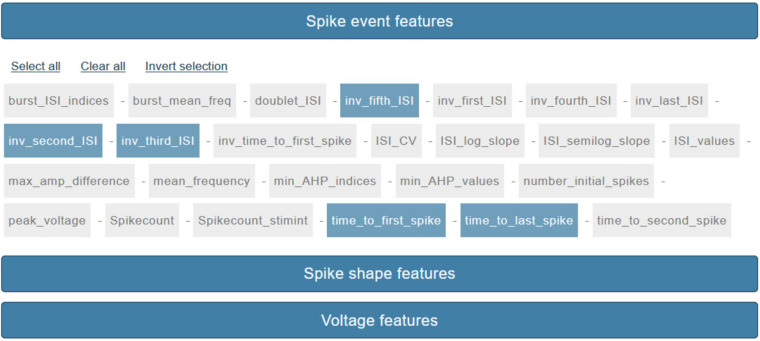
Feature selection panel. About seventy features are currently available for selection via a point-and-click interface. Features are grouped by three categories: “Spike event features,” “Spike shape features,” “Voltage features.”

The last step, before the actual feature extraction process is launched, consists in the feature selection (via a point-and-click interface, see Figure 4). The features that the users can select are those available and documented in the eFEL python package (except for a small number of features that require the stimulus waveform in order to be extracted) and are grouped by category (see section *Methods/eFEL*).

### Extracted Features

**FIGURE 5:**
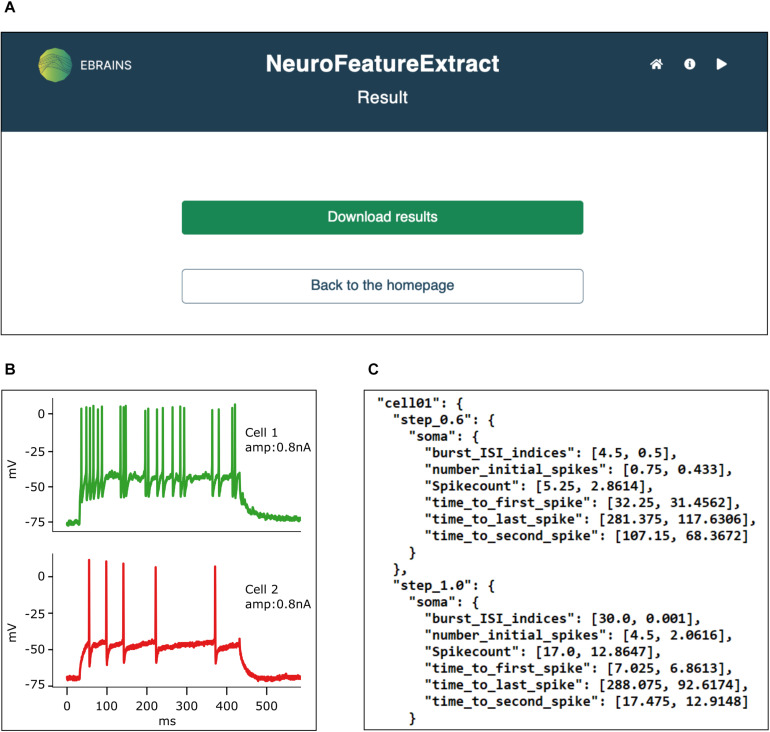
Result panel. **(A)** All results are downloadable from the web interface in a unique .*zip* file. **(B)** The traces from which the features have been extracted are printed to .*pdf* files. **(C)** Feature mean and standard deviation values are saved in appropriately formatted .*json* files, ready to be fed to the BluePyOpt neural model optimizer. Data from Migliore et al. (2018).

The feature extraction results are available for download as a .*zip* file, after the data processing is completed (see Figure 5A). The results folder contains the output files generated by the BluePyEfe Python package, which in turn leverages the eFEL library (see sections *Methods/BluePyEfe* and *Methods/eFEL*), and passed “as is” to the user. More specifically, in the main folder, a subfolder for each cell is created that contains: (1) a .*pdf* file displaying the electrophysiological traces from which the features have been extracted (see Figure 5B); (2) a .*pdf* file displaying the corresponding stimulus traces; (3) a .*pdf* file containing the plot of the mean values and standard deviations of the extracted features (computed on sets of traces recorded upon delivery of the same stimulus); (4) a *features.json* files containing mean values and standard deviations of the computed features grouped by stimulus amplitude (see Figure 5C); (5) a *protocols.json* file where the stimulation protocols is reported in appropriately structured Python dictionaries. In addition to the results obtained for individual cells, the same files listed above are generated (and saved in the root of the results folder) where the statistics and plots have been computed after averaging among individual cells. In addition, the *all_feature_table.txt* tab-separated text file is included, where individual features extracted from individual traces are reported in tabular format. This notation provides the users with a helpful mean to compute higher order statistics on raw (i.e., not averaged) feature values. It is worth noting that the *result* files are explicitly formatted to be compatible with the neural optimization software library BluePyOpt (Van Geit et al., 2016). This Python tool (freely available online on PyPi^26^ and GitHub^27^) allows to optimize neural models via genetic algorithms and is being extensively used by neuroscientists for optimizing different types of neural models against experimental data (Migliore et al., 2018; Masoli et al., 2020; Rizza et al., 2021). The NFE is integrated in the Hodgkin-Huxley Neuron Builder web application that provides the community with an easy-to-use interface able to facilitate the optimization workflow.^28^

**FIGURE 6:**
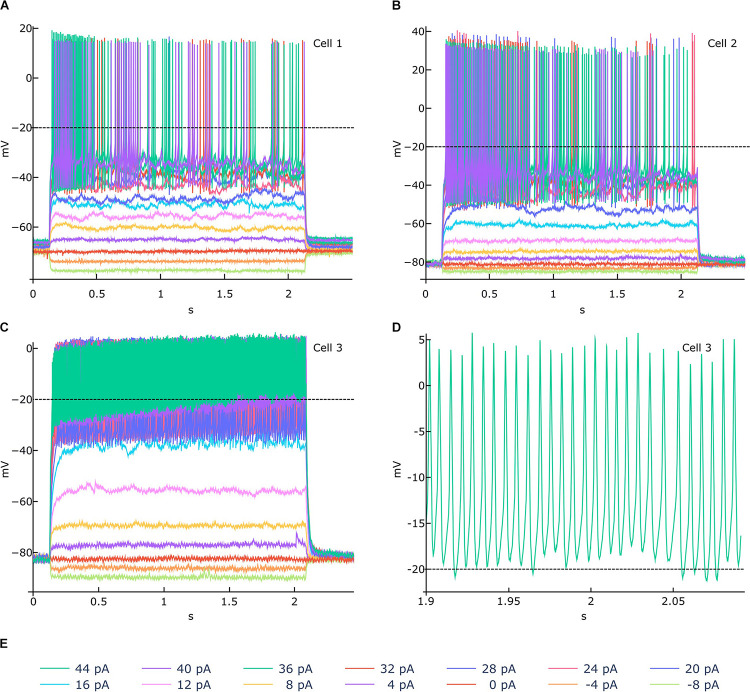
Feature extraction parameter tuning. **(A–C)** Example of electrophysiological recordings (related to 3 different cells) acquired upon delivery of the 14 stimuli displayed in **(E)** (amplitudes from -8 to 44 pA, at 4 pA intervals). In **(A,B)** a −20 mV spike detection threshold (the default value) is appropriate for identifying all the action potentials. In **(C)**, an activity drift is observed for stimuli greater than 16 pA. If a −20 mV threshold is used in this case, the spike detection process would fail to recognize a significant number of action potentials, in that they occur above the threshold itself, as shown in **(D)** (zoomed activity chunk for the 44 pA recording of Cell 3). For such a scenario, the NFE trace visualization panels (see Figure 3) allows to identify an appropriate threshold that can be set through the panel dedicated to the extraction parameters tuning (see Figure 2A). Data courtesy of Prof. E. D’Angelo.

In order to demonstrate the usefulness of the functionalities available in the NFE, we selected three traces from the HBP dataset for feature extraction and went through the entire extraction pipeline. These recordings have been acquired from mouse cerebellar granule cells via patch clamp experiments and are shown in Figure 6. While the signal-to-noise ratio of all experiments is high and action potentials seem to be unambiguously detectable, if only upon visual inspection (Figures 6A–C), the stimulus highest values induce an activity drift in one of the recordings (Figure 6C). This behavior is bound to affect the validity of the extracted feature values, in that the default spike detection threshold (i.e., -20 mV) would fail to correctly identify a collection of action potentials occurring jointly with the drift, for high stimulus amplitudes (Figures 6C–E). To address this issue, the users can zoom in and out the traces, via the trace visualization interactive panels (Figure 4), in order to identify a suitable threshold able to guarantee a correct spike detection for all the recordings. Then, via the Feature Extraction Parameters panel (Figure 2A), the appropriate threshold can be set before the feature extraction is triggered.

In order to quantify the impact of a wrong spike detection threshold setting, we extracted the mean and standard deviation of the Burst Number, Mean Frequency, Spike Count and Action Potential Width features, for the traces shown in Figure 6 (we only considered the 44pA stimulus amplitude for demonstration purposes) upon adoption of two different thresholds. These values are reported in Table 1.

**TABLE 1:** Mean and standard deviation values of four features extracted from the traces shown in Figure 6 (for stimulus amplitude equal to 44pA).

Feature name	Spike detection threshold: −20 mV			Spike detection threshold: −5 mV		
	**Mean**		**SD**	**Mean**		**SD**
Spike count	135.0		96.2	153.7		122.6
Mean frequency (Hz)	70.1		48.2	78.7		60.4
Burst number	7.7		2.5	6.3		4.1
Action potential width (ms)	1.7		1.4	0.7		0.2

*All values are strongly affected by the computed number of action potentials, namely the activity Spike Count (a -20 mV threshold fails to detect a significant number of spike events). Interestingly, both the mean and standard deviation values of the action potential width is much higher when a -20 mV threshold is adopted. This is explained by the fact that one or multiple portions of the recorded firing activity entirely occur above the spike detection threshold and are consequently read as individual action potentials with particularly large -and not biophysically plausible- width.*

## Discussion

In the framework of the HBP (Amunts et al., 2016), we have developed an online tool for the extraction of electrophysiological features from either recorded or simulated neural activity. The NFE is a web application freely accessible on the internet that provides the community with a user-friendly interface for: (1) visualization and selection of neural signals; (2) selection of electrophysiological features of interest; (3) feature extraction.

The NFE provides the users with a set of recordings contributed by the HBP consortium and partners that are, or are being, integrated in the EBRAINS research infrastructure data management engine (i.e., the EBRAINS KG). In addition, a unique feature of the NFE is the possibility to upload user’s own data files to be analyzed individually or in conjunction with the recordings selected from the EBRAINS available dataset. The application currently accepts two different file formats for the upload: (1) .*abf* files (i.e., axon binary format), which needs to be provided together with a metadata file and (2) .*json* files containing all the data and metadata required to perform the feature extraction procedure. In particular, the latter format has been designed so as to be created by computational neuroscientists or experimentalists with basic knowledge of software programming. In fact, accepted .*json* files are text files that contain neural traces and related info (e.g., stimulus amplitude, stimulus start and end time, data units) in appropriately structured dictionaries, easily accessible by the underlying feature extraction software package, the BluePyEfe, which has been purposefully extended to support this data format (see section “Methods”).

Another key feature of NFE is the varied repertoire of the generated outputs. Not only the .*json* files needed to be fed to the BluePyOpt neural model optimizer are produced, but also the features extracted from individual traces are stored (upon selection of the relevant option) in a properly formatted .*txt* file, easily manageable for further processing.

Finally, usability is made simpler by a dedicated documentation and an interactive video tutorial that guide the users through the entire feature extraction workflow.

While the NFE already offers a self-consistent means for data analysis on electrophysiological traces, the tool is being currently extended with further features.

For example, the interaction with the EBRAINS KG is being made tighter: a dedicated engine that periodically verifies the availability of suitable data for the feature extraction procedure is currently under development. Not only will this functionality allow to check in a (quasi) real-time manner which data have been added to the KG and whether these data are eligible for feature extraction (i.e., they refer to step current experiments and contain the needed metadata), but also it will perform a seamless interaction with the KG for data reading and fetching.

Also, we will further develop the NFE so as to allow the users to keep track of the data files from which the features have been extracted, the extraction process parameters and the version of the software packages used for the entire workflow. In order to achieve this goal, we will leverage the EBRAINS provenance tracking engine and store the relevant metadata in the EBRAINS KG so as to allow the users to both fetch previous processes relevant information and use them for novel feature extraction procedures or for feature extraction validation.

## Data Availability Statement

Publicly available datasets were analyzed in this study. This data can be found here: https://hbp-bsp-hhnb.cineca.it/efelg/docs/dataset/.

## Author Contributions

MM and LB contributed to conception and design of the study and wrote the first draft. LB, RS, DC, and MM chose the methodology to adopt. LB, RS, and DC developed the software. MM supervised the study. All authors contributed to manuscript revision, read, and approved the submitted version.

## Conflict of Interest

The authors declare that the research was conducted in the absence of any commercial or financial relationships that could be construed as a potential conflict of interest.

## Publisher’s Note

All claims expressed in this article are solely those of the authors and do not necessarily represent those of their affiliated organizations, or those of the publisher, the editors and the reviewers. Any product that may be evaluated in this article, or claim that may be made by its manufacturer, is not guaranteed or endorsed by the publisher.

## Footnotes

1
https://senselab.med.yale.edu/ModelDB/

2
https://www.dandiarchive.org/

3
https://zenodo.org

4
https://datadryad.org/

5
https://figshare.com

6
https://nwb.org

7
https://github.com/NeurodataWithoutBorders/nwb-jupyter-widgets/

8
http://nwbexplorer.opensourcebrain.org

9
https://alleninstitute.org/

10
https://kg.ebrains.eu/

11
www.ebrains.eu

12
https://hbp-bsp-hhnb.cineca.it/efelg/

13
www.djangoproject.com

14
www.cineca.it

15
www.openstack.org

16
www.python.org

17
www.nginx.com

18
https://uwsgi-docs.readthedocs.io/

19
https://github.com/cnr-ibf-pa/hbp-bsp-hh-neuron-builder/tree/master/efelg

20
https://pypi.org/project/efel/

21
https://github.com/BlueBrain/eFEL

22
https://efel.readthedocs.io/en/latest/

23
https://pypi.org/project/bluepyefe/

24
https://github.com/BlueBrain/BluePyEfe

25
https://hbp-bsp-hhnb.cineca.it/efelg/docs/

26
https://pypi.org/project/bluepyopt/

27
https://github.com/BlueBrain/BluePyOpt

28
https://hbp-bsp-hhnb.cineca.it/hh-neuron-builder/
